# The Micrococcus of Acute Rheumatism

**Published:** 1903-02-14

**Authors:** 


### THE MICROCOCCUS OF ACUTE RHEUMATISM.

We are told that at the present day it is hardly necessary to discuss the question of the infectiveness of acute rheumatism. The centre of interest now moves forward to the question of the characters of the micro-organism by which the disease is caused. Discussing this matter in the *Practitioner*, Dr. Ainley Walker begins by showing how in its clinical and hygienic aspects acute rheumatism presents all the characters of an infective illness. It is, he says, to some extent endemic in certain areas; it exhibits at times a tendency to epidemic prevalence; and it presents the stigmata of an attenuated septicæmia. That it is due to an irritant circulating in the blood is evidenced by the occurrence of pyrexia, rapid anæmia, erythemata and purpura, polyarthritis, pericarditis, myocarditis, endocarditis, often albuminuria, and at times pleurisy, pneumonia, and nephritis; and that this irritant is of microbic origin is rendered probable by the frequency with which the onset of the symptoms can be associated with an antecedent pharyngeal inflammation, the familiar rheumatic tonsillitis. As to the nature and relationships of this microbe Dr. Ainley Walker shows that one and the same micro-organism is obtainable from acute rheumatism, from malignant endocarditis, and from chorea; and, on the other hand, that the organism isolated from a simple rheumatism may produce in animals malignant endocarditis, and probably at times chorea also.

Until lately, owing to the variety of organisms discovered by different observers, the tendency has been to attribute the disease to a variety of agents acting upon a particular type of tissue-soil, or to regard it as an infection by attenuated pyogenetic staphylococci or streptococci; while certain of its features were attributed to mixed infections. Attention has, however, within the last few years been definitely centred upon an organism described by Apert and Triboulet in 1898, which has since been carefully examined by Paine and Poynton, who have found the organism microscopically in nearly all the lesions of acute rheumatism, and regard it as a specific cause of that disease. With this conclusion Dr. Ainley Walker, as the result of a re-investigation of the whole subject, agrees, holding that a particular micro-organism and no other is the causal agent in the production of acute rheumatism. Whether, however, this particular coccus is merely an ordinary streptococcus or is something specific (on which hangs the whole question whether rheumatism is a specific disease or merely an attenuated streptococcic septicæmia, as some hold), still remains unproven, although clinically there is everything and bacteriologically there is much in favour of its being specific. Once prove that the rheumatic micrococcus can be with certainty distinguished from all other streptococci, and we shall on the one hand possess a means by which rheumatism may be definitely distinguished from the vast array of conditions by which clinically it is imitated, while on the other hand the field will lie open for the discovery of some specific therapeutic agent by which the disease may be brought under control.

